# Coverage and Influencing Determinants of Influenza Vaccination in Elderly Patients in a Country with a Poor Vaccination Implementation

**DOI:** 10.3390/ijerph14060665

**Published:** 2017-06-20

**Authors:** Maria Ganczak, Karolina Gil, Marcin Korzeń, Marta Bażydło

**Affiliations:** 1 Department of Epidemiology and Management, Pomeranian Medical University, 71-210 Szczecin, Poland; 2 Students’ Scientific Association, Pomeranian Medical University, 71-210 Szczecin, Poland; gil.karolina.anna@gmail.com; 3 Department of Methods of Artificial Intelligence and Applied Mathematics, West Pomeranian University of Technology, 71-210 Szczecin, Poland; mkorzen@wi.zut.edu.pl; 4 Department of Public Health, Pomeranian Medical University, 71-210 Szczecin, Poland; marta.bazydlo@pum.edu.pl

**Keywords:** influenza, vaccination, coverage, elderly patients, determinants

## Abstract

The seasonal influenza vaccination uptake of the elderly in Poland is one of the lowest in Europe. *Objective*: to assess the vaccination coverage and influencing determinants in patients ≥65 years of age. *Methods*: A cross-sectional study was conducted (November 2015–April 2016) among consecutive patients admitted to a municipal hospital located in the city of Szczecin, North-west Poland. Patients completed researcher-administered, anonymous questionnaires on socio- demographic data/factors related to the vaccination. *Results*: The response rate: 92.0%. Among 230 patients (79.6% women, median of age 69 years, range 65–89) who agreed to participate, 34.8% (95% Confidence Interval: 28.6–41.0%) were vaccinated. About 15.7% of respondents had not previously heard about the vaccination; 41.3% of those who stated they were vaccinated or planned on being vaccinated the following year, compared to 19.3% of respondents who stated they were not currently vaccinated (*p* < 0.001). A multivariable regression analysis revealed that patient factors, such as younger age (Odds Ratio, OR = 7.69), living in the urban area (OR = 7.69), having comorbidities (OR = 2.70), having a vaccinated family member (OR = 3.57), and being informed about vaccination (OR = 5.00) were each associated with greater odds of being immunized. Willingness for vaccination the next year was strongly associated (OR = 8.59) with vaccination status. *Conclusions*: The influenza vaccination uptake in the elderly population in Poland is disturbingly low. Improved education strategies are needed to increase the uptake. Vaccinated respondents are more likely to plan on being vaccinated the following year. Future interventions related to maximizing vaccination coverage should be more tailored, focusing especially on older patients living in rural areas.

## 1. Introduction

Influenza is a common infectious disease of the respiratory system. According to the World Health Organization (WHO), between 330 million and 1.575 billion individuals worldwide suffer from influenza and influenza-like viruses yearly, with 0.5 and 1 million deaths [1]. In the European Union (EU), seasonal influenza causes 40–50 million symptomatic cases each year, and 15,000–70,000 EU citizens die of causes associated with influenza [2]. However, the real incidence of the disease is underestimated, as a considerable portion of the cases are not registered. According to the Polish National Institute of Hygiene data, a total of 3,164,405 confirmed and suspected cases of influenza were reported in 2014, an incidence of approximately 8219 per 100,000 population [3]. Of note, most of the reported hospitalized, laboratory-confirmed cases occurred in people aged 65 years or more. Influenza infection in this subgroup is associated with higher morbidity and worse outcomes [4,5].

Immunization is an important and cost-effective public health intervention to reduce influenza morbidity and mortality [6]. Annual vaccination of older adults and other high risk groups is the most effective measure for reducing morbidity and mortality associated with infection [7]. For community-dwelling elderly, the adjusted analyses from cohort and case control studies in the Cochrane review show reductions in the risk of hospitalizations for influenza, pneumonia, respiratory or cardiac diseases, and for all-cause mortality and death from influenza and pneumonia [6]. The Advisory Committee on Immunization in the US, as well as various expert panels, have recommended a routine vaccination against influenza for all individuals above the age of 6 months to old age [8]. In Poland, influenza vaccination is among recommended vaccinations in the National Immunization Program; however, is not reimbursed via national health insurance. The guidelines recommend vaccination of children 0.5–18 years, patients over 55 years, those chronically ill, and those who are immunosuppressed (clinical indications), as well as healthcare workers and those exposed to contact with a large number of individuals, including persons working in education, trade, and transport sectors (epidemiological indications) [9]. The average cost of the vaccine is approximately 7 USD.

The WHO and the Council of the EU recommend the level of vaccination rates among the elderly to be 75%. This would significantly reduce the spread of influenza, decrease the number of deaths from the disease, and reduce the direct and indirect costs generated during an epidemic [10,11]. However, recent reports suggest that influenza vaccination coverage in Poland in those aged ≥65 years is far from satisfactory (7.7%) [3], and is in fact one of the lowest in Europe [12]. Therefore, several initiatives were undertaken by local governments in recent years to improve influenza vaccination uptake among elderly patients, including a provision of free of charge vaccinations [13]. Unfortunately, these programs have been limited and have not succeeded in achieving WHO goals regarding vaccination coverage.

## 2. Objective

A variety of studies have explored the determinants of vaccination uptake among elderly patients in countries where the coverage in this subgroup is optimal and suboptimal [5,10,14,15]. However, data coming from Poland, a country in which the uptake is one of the lowest in the EU, are rather scant [13,16,17]. Therefore, the objective of this study was to assess vaccination coverage and influencing determinants of seasonal influenza vaccination in hospitalized Polish patients ≥65 years of age. Such evidence could be used to develop optimal strategies to improve vaccination uptake in this at-risk population.

## 3. Materials and Methods

### 3.1. Design and Setting

A cross-sectional study was conducted from November 2015 to April 2016 among consecutive patients admitted to one municipal hospital in the city of Szczecin, in Northwestern Poland.

### 3.2. Study Population and Sampling

The following eligibility criteria needed to be met in order to participate in the study: age ≥65 years, ability to give informed consent, lack of comorbidities that could prevent effective communication, and patient agreement to take part in the study. The patient interviews were conducted immediately after they had been admitted to the hospital.

### 3.3. Study Instrument

A researcher-administered anonymous questionnaire was designed by the study team using a literature review [13,14,17,18,19]. In case of any queries, participants were provided with relevant information, as well as any explanations that would allow them to better understand and correctly answer the questions. Relatives were also contacted if there was such a need to verify patients’ vaccination status. The questionnaire was pilot-tested on 20 randomly selected elderly patients admitted to one hospital ward (results included in the study). It consisted of 32 multiple-choice questions with one or more pre-defined answers. It included questions about: (1) socio-demographic measures: age, gender, place of residence, education level, economic status, source of income, living arrangements; (2) self-assessment of health status, chronic diseases; (3) influenza vaccination uptake in 2015/2016 season, past vaccination history, vaccination of family members; (4) beliefs about influenza vaccination, reasons for not receiving the vaccination; and (5) previous information on influenza vaccination.

At the Pomeranian Medical University, there is no requirement for ethics committee approval for studies similar to ours. Nevertheless, before fulfilling a questionnaire, the purpose of the study was explained to every patient, who was then assured that he/she would not be identified in any presentation or publication. To protect the confidentiality of the subjects, completed questionnaires were stored in a locked filing cabinet, and computer data were password protected and accessible only to the four study investigators.

### 3.4. Statistical Analysis

Data analysis was carried out with STATISTICA (PL Version 7.1., StatSoft Inc., Kraków, Poland, 2005) and R (R version 3.x) software [20]. Our primary outcome variable was seasonal influenza vaccination and we aimed to identify variables associated with this outcome. Bivariate analysis assessed demographic characteristics: age (≤70 years/>70 years), gender, residency (urban/rural), education level (basic-secondary/high), socioeconomic status (high/poor), source of income (employment/pension-disability benefit-dependent (no income), living arrangements (alone/with a family member-nursing home), together with patient health status (very good/poor-very poor; comorbidities: yes/no), vaccination of a family member (yes/no-cannot recall), received information about influenza vaccination (yes/no), willingness to be vaccinated in the next year (yes/no-do not know), associated with an outcome variable. For categorical (binary) variables, as described above, groups were compared using the chi square and Fisher tests. To build a logistic regression model [21], the set of predictors was used and a stepwise backward selection procedure was performed with the help of the R MASS package [22]. Final associations between predictors and the outcome adjusted for covariates were measured with the use of coefficients of a logistic regression model. Coefficients for binary variables are equal to the natural logarithm of the odds ratio.

## 4. Results

### 4.1. Demographic

The analysis included 230 of the 250 questionnaires distributed (a response rate of 92%). Patients’ demographic characteristics are presented in Table 1. The age of respondents ranged from 65 to 89 (median 70) years; women accounted for 79.6%. The majority of patients (92.2%) were from urban areas and had finished education at the high school level (86.1%). Almost two thirds (63.0%) self-assessed their socio-economic status as good/satisfactory; 59.1% were retired, 62.6% lived with family. More than one third of the patients (39.1%) self-assessed their health status as poor; 83.9% reported having chronic diseases.

### 4.2. Vaccination Status

More than one third of patients (*n* = 80, 34.8%; 95% confidence interval (95% CI): 28.6–41.0%) was vaccinated against seasonal influenza, 60.4% (*n* = 139) were not vaccinated, and 4.8% (*n* = 11) could not recall. Of those vaccinated 66.3% (*n* = 53) reported that this was not their first influenza vaccination, for 12.5% (*n* = 10) it was the first, and 21.2% (*n* = 17) could not recall. For more than one third of patients (35.0%; *n* = 28), the vaccine was free of charge. Of 80 respondents who stated that they were vaccinated, 41.3% (*n* = 33) planned on being vaccinated the following year, compared to only 19.3% (29/150) of respondents who stated they were not currently vaccinated (*p* = 0.0004).

About one in four (23.5%) of respondents reported that a family member was vaccinated against seasonal influenza (Table 1).

### 4.3. Sources of Information and Reasons for Influenza Vaccination

The majority of respondents (80.9%; 186/230) were informed about the influenza vaccination, 15.7% (36/230) were not, and 3.4% (8/230) could not recall.

The main source of information on vaccination was a family doctor (44.8%; *n* = 103), followed by television (36.1%; *n* = 83), friends (19.1%; *n* = 44), brochures (18.7%; *n* = 43), newspapers (17.0%; *n* = 39), nurse (13.5%; *n* = 31), internet (4.8%; *n* = 11), and other (2.2%; *n* = 5); this was a multiple-choice question.

Most vaccinated patients (70.0%; *n* = 56) reported that their decision regarding immunization was based on the recommendation of a family doctor, for 35.0% (*n* = 28) it was family/friends, then internet (6.25%; *n* = 5), a nurse (6.25%, *n* = 5), another physician (2.5%; *n* = 2); for 7.5% (*n* = 6) of respondents it was their own decision.

**Figure 1:**
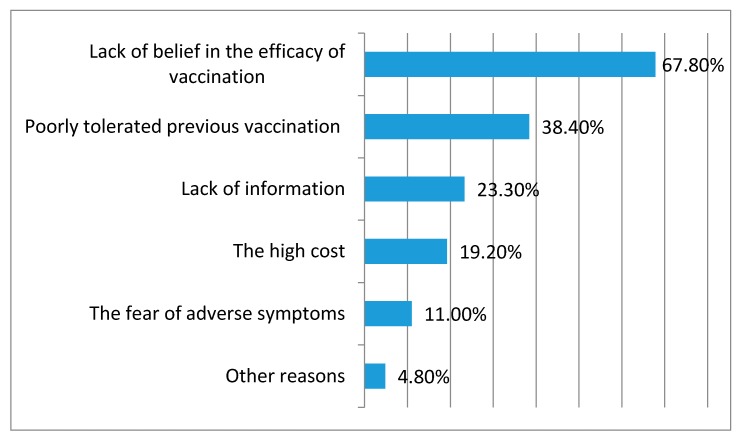
Reasons for not receiving the influenza vaccine among the elderly patients. Szczecin, Poland, 2015–2016; *n* = 230.

The main explanation for not being vaccinated was lack of belief in vaccination efficacy (67.8%; *n* = 100), followed by a poorly tolerated previous vaccination (38.4%; *n* = 58), lack of information (23.3%; *n* = 35), high cost (19.2%; *n* = 29), and fear of adverse symptoms (11.0%; *n* = 17); see Figure 1.

### 4.4. Beliefs about Influenza Vaccination

Individuals were asked about their beliefs regarding the reasons for which patients ≥65 years of age refuse influenza vaccination. More than a third of respondents (36.5%) believed that the main reason to refuse immunization is the lack of vaccine effectiveness, for 27.8% it was the lack of time, for 17.8% it was the high cost, and for 16.1% it was the complicated vaccination procedure.

### 4.5. Determinants Related to Vaccination

Detailed information on the associations between patient characteristics and the influenza vaccination rate is presented in Table 1.

An analysis of the data revealed that younger patients (≤70 years) were vaccinated significantly more often (*p* = 0.04) than those >70 years. The vaccination uptake among patients living in urban areas was significantly higher than among those living in rural areas (*p* = 0.04). In patients who reported having a chronic disease and those who described their health status as poor/very poor, the percentage of vaccinated individuals was significantly higher than those who did not (*p* = 0.04 and *p* = 0.02 respectively).

There were no statistically significant differences between patients vaccinated and un-vaccinated regarding gender (*p* > 0.39), education level (*p* = 0.55), economic status (*p* = 1.00), source of income (*p* = 0.12), and accommodation (*p* = 0.06).

Patients who received information about vaccination were significantly (*p* < 0.0001) more often immunized than those who did not.

All variables significant at the bivariate level were then entered into a stepwise multivariate model. It revealed that younger age (odds ratio (OR) = 7.69), living in an urban area (OR = 7.69), having comorbidities (OR = 2.70), having a vaccinated family member (OR = 3.57), and being informed about vaccination (OR = 5.00) were each associated with greater odds of being immunized. Willingness to be vaccinated the next year was strongly associated (OR = 8.59) with vaccination status (Table 2).

**Figure 2:**
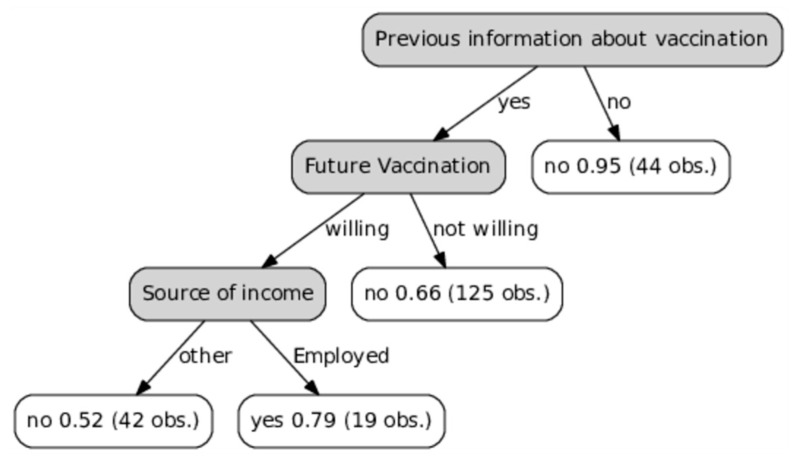
Decision tree illustrating how previous information about vaccination determines vaccinating an elderly patient against seasonal influenza.

As previous information on vaccination was a strong determinant of vaccinating an elderly patient against influenza, a decision tree was used as a visual and analytical decision support tool. As illustrated in Figure 2, almost none (95%) of patients who had not received influenza vaccination information (right arm) were vaccinated. Moreover, for the patients who had received information on influenza immunization (left arm), this was a strong determinant for planning on being vaccinated the following year: two thirds (66%) of those not willing to be vaccinated during the next season were not vaccinated.

## 5. Discussion

### 5.1. Overview of Results

Our findings indicate that only around one third of elderly patients admitted to a Polish municipal hospital were vaccinated against seasonal influenza. The main reason for not being vaccinated was a lack of belief in the efficacy of vaccination. Patient-related factors, such as younger age, living in an urban area, having comorbidities, having a vaccinated family member, as well as being informed about vaccination were each associated with higher odds of immunization. Willingness to be vaccinated the next year was strongly associated with vaccination status.

### 5.2. Vaccination Uptake

The results of this study show that there is still a long way to fulfill the WHO and the Council of the EU recommendations regarding seasonal influenza vaccination rates among the elderly in Poland.

It is estimated that only about 2.5% of Poles were vaccinated against seasonal influenza in 2014 [3,12]. Even more worrying is the fact that vaccine uptake in such a high-risk group as the elderly remains low when compared to other European countries. Blank et al. studied vaccination coverage rates in the elderly in 11 European countries during two consecutive influenza seasons (2006/07 and 2007/08), and found the lowest (13.9%) coverage rates in Poland and the highest in the United Kingdom (70.2%) [10]. Vaccination uptake in this study, conducted 10 years later, was higher than reported above, however it was still not satisfactory and far below the WHO and the Council of the EU recommendations.

A possible explanation for the poor vaccination rates in the elderly in Poland could be the lack of influenza vaccine provision within public health insurance and the lack of reimbursement for healthcare practitioners to administer the vaccine.

The cost of the influenza vaccine itself was reported as a critical barrier regarding vaccination [14]. Some studies from countries where patients have to pay for the vaccine found cost to be an important determinant regarding the uptake [23]. Fedson et al. gathered information on influenza vaccine distribution in 18 developed countries; 11 provided reimbursement for vaccination through national or social health insurance. These countries tended to have higher levels of vaccine use [24]. In Poland patients do not have insurance coverage for the vaccine. In addition, initiatives undertaken by local governments, such as a provision for free of charge vaccinations among elderly patients, are limited. From 114 communities in the West Pomeranian region, in which our study was carried out, only 18 provide seasonal influenza vaccination for senior citizens at no cost [25].

For the elderly patients in this study, the main reason for not being vaccinated was a lack of belief in the vaccination efficacy; this was also reported by others [13,15,17,23]. Those who refused were reported to have no confidence in the immunization [14].

### 5.3. Factors Related to Vaccination

Factors increasing the risk of an elderly person not taking the seasonal influenza vaccination identified in this study are complex and similar to those affecting vaccination coverage in other countries with sub-optimal uptake [10,14,15,26,27,28,29].

Vaccine hesitancy among the elderly may be the result of several factors. Firstly, the vaccination should be repeated annually; this requirement is not easy to understand and difficult to fulfill for individuals with various comorbidities, disabilities, or those living alone, etc. Some influenza- specific myths which still exist are difficult to dispel, specifically in this age group. Finally, vaccine effectiveness varies annually and is often low, which is another factor that might influence an immunization decision [15].

### 5.4. Structural Patient-Related Determinants

Surveys performed in several countries found that older age (≥75 years) has been associated with higher vaccination rates [14,29,30,31]. However, our study revealed that younger patients were almost eight times more likely to receive the influenza vaccine. The dominance of younger patients among those vaccinated can be explained by the fact that older age is often associated with imperfect functional status, which may negatively influence the likelihood of vaccine uptake since access might depend on transportation or assistance [14].

Place of residence may determine ease of access to vaccination. Patients from urban areas had almost an eight times higher likelihood of influenza vaccination than those from rural settings. Data on the differences between vaccination uptake in rural and urban areas are inconsistent and depend on country and health system characteristics [14]. As an example, De Andres et al. found that living in a town with more than 10,000 inhabitants increased the likelihood of vaccination among senior citizens [29]. In contrast, other studies reported that urban settings had a lower likelihood of influenza vaccination than rural towns [27,32,33]. Further research is needed to provide evidence regarding the association of place of residence with influenza vaccination uptake in the elderly.

In this study, the logistic regression analysis revealed that having chronic diseases was associated with a three times higher chance for having influenza vaccination when compared to not having a chronic disease. The possible explanation of this finding is that because chronic diseases are an indication for immunization against influenza, the frequency of vaccination is likely to be higher with their presence. Our results support the previous reports on influenza vaccination in the elderly [16,28,29,30,31,32,33].

Similar to other studies conducted in Poland [17] and abroad [28,29], the bivariate analysis performed in this study revealed that patients who assessed self-health status as “poor” were more likely to be vaccinated in comparison to patients with “good” health status. Possibly patients who perceived themselves as being in poor health were aware of the increased probability of acquiring influenza or experiencing severe influenza complications, which could influence their decision regarding immunization.

Consistent with other studies [23,32,34,35], our study confirmed that a recommendation from family may positively influence vaccine acceptance. Elderly patients tend to trust family members’ advice. As an example, one senior Chinese patient interviewed by Kwong et al. stated “My daughter told me about it, I had it done based on her recommendation. I trust my daughter” [23].

We found that a willingness for vaccination the next year was strongly associated (OR = 8.6) with vaccination status; 41% of respondents who stated that they were vaccinated also planned on being vaccinated the following year, compared to only 19% of respondents who stated that they were not currently vaccinated. This was also reported by others [18,35]. If an individual has a positive initial experience regarding influenza vaccination, he/she is more likely to request the vaccination during the next season and then to do it regularly [14].

Important sources of information for our patients were medical staff, mainly a family doctor, television, and friends, which is in line with other studies [16,17,32,35,36]. Furthermore, most vaccinated patients reported that their decision was based on a family doctor recommendation. This was also reported by others [10,13,16,36]. We found that receiving information on influenza immunization was associated with a five times higher chance of being vaccinated; almost none of the patients who had not received such information were immunized. Furthermore, as illustrated in Figure 2, receiving relevant information was a strong determinant for planning on being vaccinated the next year. Similarly, Nessler et al. also reported that patients from Polish primary care clinics who had received sufficient influenza vaccination education from their healthcare provider had significantly higher vaccination rates [16]. In another Polish study conducted by Łukomska et al., almost half of unvaccinated patients (48%) believed that the lack of sufficient information might be the main reason influencing poor vaccination rates [17]. Notably, one in six elderly patients in this study had not heard about the influenza vaccination.

### 5.5. Implications for Immunization Policy

Future interventions to improve vaccination uptake among the elderly in a country with a poor vaccination implementation, such as Poland, should include governmental help toward making it free of charge or partly refunded. According to Brydak et al., implementing a vaccination program in Poland in which the influenza vaccination would be fully reimbursed by National Health Insurance for people aged ≥65 years would be a very cost-effective strategy compared to the current situation, with an incremental cost-effectiveness ratio of 7303 USD/quality-adjusted life year (QALY); this is below the yearly gross domestic product per capita [37].

Our study revealed that the majority of unvaccinated elderly patients were not confident of influenza immunization effectiveness. Therefore, interventions should concentrate on strategies which encourage an increase in confidence and recognize it as a preventive measure. Patients should be informed that even during seasons when vaccine effectiveness is reduced, vaccination can offer substantial benefit and might reduce the likelihood of severe outcomes such as hospitalization and death [38]. The wider adoption of quadrivalent influenza vaccine in immunization programs may help to address the problem of influenza B lineage mismatch [39], and thus gives promise to increase vaccine effectiveness which may in turn restore confidence.

The results of the study showed that being informed about vaccination was associated with greater odds of being immunized, and it also positively influenced plans on being vaccinated the following year. Thus, providing elderly patients with detailed information regarding influenza immunization is of a great importance. The necessity of vaccination for this vulnerable group, as well as its benefits, should be thoroughly explained, together with information regarding when and where the vaccine can be obtained. Most vaccinated elderly patients reported that their decision regarding immunization was based on the recommendation of a family doctor, however, a physician was the source of information for less than half of the studied patients. Therefore, family doctors should maximize their efforts to play a more active role in immunization communication and delivery.

## 6. Limitations

This study has a number of limitations. The sample size is relatively small, so the power required for our analyses may be limited. Secondly, these findings do not necessarily apply to some other hospitals in the region and in the country, therefore further studies at a national level would be of a great value. In addition, only hospitalized patients were surveyed. As to the validity and reliability of studies that use a questionnaire as a research tool, it has been shown that respondents sometimes want to give “right” answers [40], which could likely result in a possible underestimation of the proportion of non-immunized patients. However, the fact that questionnaires were completed by an interviewer might result in social desirability bias. Moreover, recalling information about the risk factors or previous immunization could introduce a recall bias. Finally, the cross-sectional study used for the purpose of this survey is perceived as less powerful for evaluating risk factors than other analytical study designs [5]. Nevertheless, the methodology has been successfully used in recent years to evaluate determinants influencing immunization in the elderly because of its simplicity and low cost [10,14,15].

## 7. Conclusions

The influenza vaccination uptake in the elderly in Poland needs to be improved. Similar to other countries [14,15,18,19], there is no single element influencing influenza vaccination among this group in Poland [1,13,16,17], but rather a variety of factors, which play different roles depending on the region, province, or community. Therefore, national strategy should concentrate on evidence- based public health practices according to the local epidemiologic situation, and on the results of thorough research with the use of scientifically approved methods. Future interventions related to maximizing vaccination coverage should be more tailored, focusing especially on providing patients with relevant information on vaccine effectiveness, together with advice and support from professionals, to enhance vaccination coverage among vulnerable sub-groups identified in this study which are particularly difficult to reach, such as older patients living in rural areas. Funded vaccination programs accompanied by high awareness in the elderly are crucial to increasing influenza vaccination uptake.

## Figures and Tables

**Table 1:** Characteristics of elderly patients vaccinated and unvaccinated against influenza in Szczecin, Poland, 2015–2016; *n* = 230.

Variable	Number of Respondents *n* (%)	Vaccinated *n* (%)	Unvaccinated *n* (%)	*p*
*Gender*				
women	183 (79.6)	61 (76.2)	122 (81.3)	0.39
men	47 (20.4)	19 (23.8)	28 (18.7)	
*Age (Years)*				
65–70	125 (54.3)	51 (63.7)	74 (49.3)	0.04
71–80	66 (28.7)	14 (17.5)	52 (34.7)	
>80	39 (17.0)	15 (18.8)	24 (16.0)	
*Residency*				
town with <50,000 inhabitants	22 (9.6)	6 (7.5)	16 (10.7)	0.04
town with 50,000–150,000 inhabitants	46 (20.0)	24 (30.0)	22 (14.6)	
town with >150,000 inhabitants	144 (62.6)	48 (60.0)	96 (64.0)	
rural area	18 (7.8)	2 (2.5)	16 (10.7)	
*Education Level*				
primary	16 (7.0)	4 (5.0)	12 (8.0)	0.55
vocational	73 (31.7)	26 (32.5)	47 (31.3)	
secondary	109 (47.4)	37 (46.25)	72 (48.0)	
university	32 (13.9)	13 (16.25)	19 (12.7)	
*Economic Status (Self-Assessed)*				
high	19 (8.2)	8 (10.0)	11 (7.3)	1.00
satisfactory	126 (54.8)	42 (52.5)	84 (56.0)	
low	85 (37.0)	30 (37.5)	55 (36.7)	
*Accommodation*				
living alone	79 (34.4)	34 (42.5)	45 (30.0)	0.06
with family	144 (62.6)	42 (52.5)	102 (68.0)	
private nursing/social welfare home	7 (3.0)	4 (5.0)	3 (2.0)	
*Source of Income*				
pension	136 (59.1)	40 (50.0)	96 (64.0)	0.12
employment	47 (20.4)	21 (26.3)	26 (17.3)	
disability benefit	42 (18.4)	18 (22.5)	24 (16.0)	
dependent (no income)	4 (1.7)	0 (0.0)	4 (2.7)	
other	1 (0.4)	1 (1.2)	0 (0.0)	
*Chronic Disease*				
present	193 (83.9)	73 (91.2)	120 (80.0)	0.04
absent	37 (16.1)	7 (8.8)	30 (20.0)	
*Health Status (Self-Assessed*)				
very good/good	140 (60.9)	40 (50.0)	100 (66.7)	0.02
poor/very poor	90 (39.1)	40 (50.0)	50 (33.3)	
*Vaccinated Family Member*				
yes	54 (23.5)	23 (28.8)	29 (19.3)	0.14
no/cannot recall	176 (76.5)	57 (71.2)	121 (80.7)	
*Previous Information about Vaccination*				
yes	186 (80.9)	80 (100.0)	106 (70.7)	<0.0001
no/cannot recall	44 (19.1)	0 (0.0)	44 (29.3)	
Total	230	80	150	-

**Table 2:** Logistic regression model after stepwise selection: association of vaccination against seasonal influenza with selected variables (odds ratio (OR) estimates and 95% confidence intervals (CIs)), *n* = 230; Pseudo R^2^ = 0.327, Area under the curve (AUC) = 0.802.

Variable	OR	95% CI
Age: <70 years	7.69	2.94–25.00
Urban area: yes	7.69	1.18–100.00
Comorbidities: yes	2.70	1.05–7.69
Received information on influenza vaccination: yes	5.00	1.23–33.89
Vaccinated family member: yes	3.57	1.29–11.13
Willingness to be vaccinated the next year: yes	8.59	3.27–26.50
